# Limite patológico da distância tendão patelar-sulco da tróclea utilizando imagens de ressonância magnética em pacientes com instabilidade patelar

**DOI:** 10.1055/s-0045-1809517

**Published:** 2025-06-23

**Authors:** Rubens Rosso Nadal, Vinícius Canello Kuhn, Alexandre Codevilla Teixeira, Eduardo Bervian Júnior, Osmar Valadão Lopes Júnior

**Affiliations:** 1 Instituto de Ortopedia e Traumatologia de Passo Fundo, Passo Fundo, RS, Brasil; 2 Departamento de Ortopedia e Traumatologia, Hospital São Vicente de Paulo, Passo Fundo, RS, Brasil; 3 Departamento de Radiologia Musculoesquelética, Clínica Kozma, Passo Fundo, RS, Brasil

**Keywords:** articulação patelofemoral, imagem por ressonância magnética, instabilidade articular, joelho, ligamento patelar, joint instability, knee, magnetic resonance imaging, patellar ligament, patellofemoral joint

## Abstract

**Objetivo:**

Avaliar e comparar a distância tendão patelar-sulco troclear (TP-ST) em indivíduos com e sem instabilidade patelar. Um segundo objetivo foi definir um limiar patológico de TP-ST por meio de ressonância magnética (RM) nuclear em pacientes com instabilidade patelar.

**Métodos:**

A distância TP-ST de 52 joelhos foi medida em 48 indivíduos com instabilidade patelar objetiva (grupo instabilidade) por RM. Essas medidas foram comparadas àquelas feitas em 50 joelhos de 44 indivíduos sem histórico de instabilidade patelar (grupo controle).

**Resultados:**

A distância TP-ST no grupo instabilidade (20,6 ± 5,0 mm) foi maior do que no grupo controle (11,8 ± 3,4 mm; *p* < 0,001). O valor de 15,5 mm foi determinado como limiar patológico, com acurácia de 81,4%, em exames de RM.

**Conclusão:**

Indivíduos com instabilidade patelar apresentam medidas estatisticamente maiores de TP-ST em comparação a pacientes sem instabilidade. Portanto, valores de TP-ST superiores a 15,5 mm à RM representam uma força de lateralização patológica do mecanismo extensor relacionada à instabilidade patelar.

## Abstract (en)

**Objective:**

To evaluate and compare the values of the patellar tendon-trochlear groove (PT-TG) distance in individuals with and without patellar instability. Additionally, we aimed to define a cut-off value for the pathological limit of the PT-TG through nuclear magnetic resonance imaging (MRI) in patients with patellar instability.

**Methods:**

The PT-TG distance of 52 knees was measured in 48 individuals with objective patellar instability (instability group) by MRI. These measurements were compared with those made in 50 knees of 44 individuals without a history of patellar instability (control group).

**Results:**

The PT-TG distance in the instability group (20.6 ± 5.0 mm) was greater than in the control group (11.8 ± 3.4 mm; *p* < 0.001). A value of 15.5 mm was determined as the pathological limit, with an accuracy of 81.4%, in MRI exams.

**Conclusion:**

Individuals with patellar instability have statistically higher measurements of PT-TG when compared with patients without it. Therefore, values higher than 15.5 mm for PT–TG seen in MRI exams represent a pathological lateralization force of the extensor mechanism related to patellar instability.

## Introdução

A instabilidade lateral da patela é uma doença com incidência entre 7 e 43 casos por 100 mil habitantes. 1 2 Os fatores de risco anatômicos são as principais causas dessa doença: displasia troclear, aumento da distância tuberosidade tibial-sulco troclear (TT-ST), inclinação patelar e patela alta. 3 4

A distância TT-ST é a distância entre o ponto ósseo mais profundo da tróclea femoral (sulco troclear, ST) e o ponto mais anterior da tuberosidade anterior da tíbia (TAT). É medida em tomografia computadorizada (TC) do joelho, e pacientes com valores iguais ou superiores a 20 mm, em casos de luxações patelares laterais objetivas recorrentes, são encaminhados para osteotomia da TAT para realinhamento. 4 5

Exames complementares de imagem são importantes para identificar fatores de risco para a instabilidade patelar e, assim, também para escolher os procedimentos a serem realizados. A TC do joelho é importante para avaliar a distância TT-ST de acordo com o Protocolo de Lyon. 4

De modo geral, a ressonância magnética (RM) nuclear do joelho auxilia a avaliação da cartilagem articular, dos ligamentos e do menisco. 6 Embora a TC seja o exame de escolha para medida da distância TT-ST, autores demonstraram que a RM também pode ser usada com este fim com excelente concordância inter e intraobservador. 6 7 8 Apesar disso, é importante notar que os valores medidos por ambos os métodos não são intercambiáveis. As medidas da RM tendem a ser menores do que as da TC. 7 Os valores obtidos pela RM são mais anatômicos em comparação aos da TC e representariam de forma mais fidedigna o vetor biomecânico que atua na instabilidade patelar. 8 A TC também seria dispensável por reduzir os custos e a exposição do paciente à radiação.

O presente estudo teve como objetivo avaliar a medida da distância entre o centro do tendão patelar e a cartilagem do sulco troclear femoral (TP-ST) em exames de RM de joelhos em pacientes com instabilidade patelar objetiva e compará-los aos valores de indivíduos sem instabilidade patelar, bem como definir o limiar patológico dessa distância.

## Materiais e Métodos

Em um estudo de caso-controle, avaliamos 102 imagens de RM de joelho de 92 indivíduos selecionados por amostragem aleatória simples de prontuários eletrônicos e subsequente análise em um banco de imagens.

A distância entre o centro do tendão patelar e a cartilagem do sulco troclear femoral de 52 joelhos foi medida em 48 indivíduos com histórico documentado de instabilidade patelar objetiva (grupo instabilidade). Essas medidas foram comparadas a 50 joelhos de 44 indivíduos submetidos a uma RM de joelho sem histórico clínico de instabilidade patelar (grupo controle).

Os critérios de inclusão no grupo instabilidade foram pacientes com instabilidade patelar lateral objetiva 9 documentada à anamnese e exame físico nos prontuários médicos do nosso banco de dados, além de indicações indiretas da RM. 10 11 12 13 Essas indicações indiretas poderiam ser contusão ou lesão osteocondral do côndilo femoral lateral ou faceta medial da patela, lesão do ligamento patelofemoral medial, lesão do retináculo medial em suas inserções patelares ou substância média, laceração do ventre distal do músculo vasto medial oblíquo e subluxação e inclinação patelar.

Do grupo instabilidade, excluímos pacientes com cirurgias prévias no joelho, histórico de trauma (exceto luxação patelar) e doenças degenerativas moderadas a graves da articulação. O grupo controle foi composto por pacientes submetidos à RM do joelho por suspeita de lesões ligamentares intra-articulares, lesões em menisco e dor com ausência de histórico de luxação ou sintomas patelofemorais prévios.

**Fig. 1:**
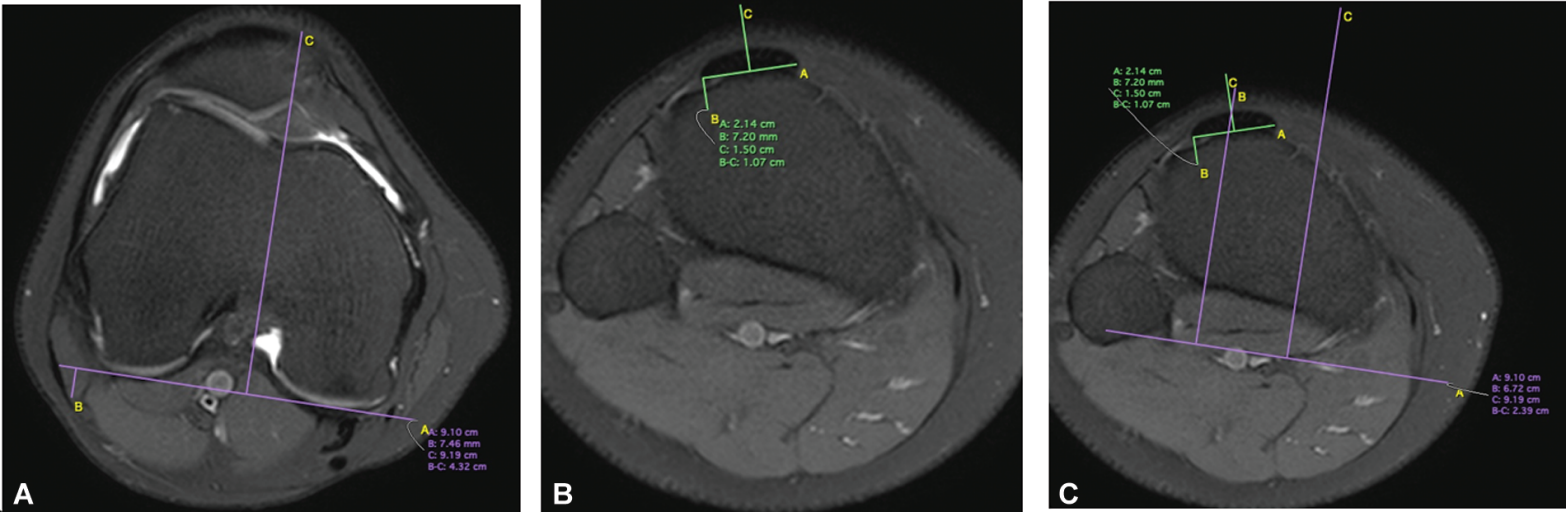
Medida da distância tendão patelar-sulco troclear (TP-ST). ( **A** ) O ponto mais profundo da tróclea, ST; ( **B** ) o centro do tendão patelar, TP; e ( **C** ) a distância entre esses dois pontos sobrepostos, TP-ST.

A RM foi realizada com o paciente em decúbito dorsal, membro em rotação neutra e extensão completa sem contração do quadríceps. Para obtenção da medida da distância entre o centro do tendão patelar e a cartilagem do sulco troclear femoral, foi utilizado o corte axial ponderado em T2 da RM, usando os parâmetros a seguir, descritos por Schoettle et al. 8 A primeira imagem craniocaudal que demonstra completamente toda a cartilagem troclear determinou o ST usando as bordas cartilaginosas dos côndilos femorais posteriores como referência. O ponto médio da inserção do TP foi definido nas imagens subsequentes distalmente em sua inserção na tuberosidade anterior da tíbia (TAT). As imagens anteriores do ST foram então transferidas para a imagem atual, que corresponde à sobreposição entre o centro do TP e o ponto mais profundo do ST. Assim, a distância TP-ST foi medida em milímetros ( Fig. 1 ).

As medidas foram realizadas por dois ortopedistas e um radiologista especialista em RM musculoesquelética com um software OsiriX DICOM Viewer (Pixmeo SARL, Genebra, Suíça). Cada RM foi avaliada pelos três avaliadores, que repetiram suas análises com um intervalo mínimo de duas semanas entre as medidas. Em nenhum momento os avaliadores tiveram acesso a qual grupo os indivíduos pertenciam, aos valores encontrados por seus pares e à sua medida anterior.

Os exames foram realizados entre abril de 2014 e setembro de 2019 e o estudo foi aprovado pelo Comitê de Ética da nossa instituição sob o número CAAE: 36188820.3.0000.5342.

### Análise Estatística

As variáveis quantitativas foram descritas como média e desvio-padrão e as variáveis categóricas como frequências absolutas e relativas. A comparação das médias entre os grupos utilizou o teste *t* de Student. A comparação entre as proporções foi feita com o teste qui-quadrado de Pearson. Para avaliação da concordância intra e interexaminadores, foram aplicados o teste t de Student para amostras pareadas e o coeficiente de correlação intraclasse (CCI). A curva característica de operação do receptor (ROC, do inglês *receiver operating characteristics* ) determinou o melhor limiar para a medida da distância TP-ST na detecção de instabilidade do joelho. O nível de significância adotado foi de 5% ( *p* < 0,05) e as análises foram realizadas no programa IBM SPSS Statistics for Windows (IBM Corp., Armonk, NY, EUA), versão 21.0.

## Resultados

A média de idade foi semelhante em ambos os grupos, de 23,3 ± 8,3 anos no grupo instabilidade e 25,4 ± 6,4 anos no grupo controle. Em relação ao lado, 53% e 48% das medidas foram feitas no joelho direito do grupo instabilidade e do grupo controle, respectivamente. Quanto ao gênero, houve diferença estatisticamente significativa entre os grupos. Os pacientes eram do sexo feminino em 65,4% dos casos no grupo instabilidade e 22% no grupo controle.

**Fig. 2:**
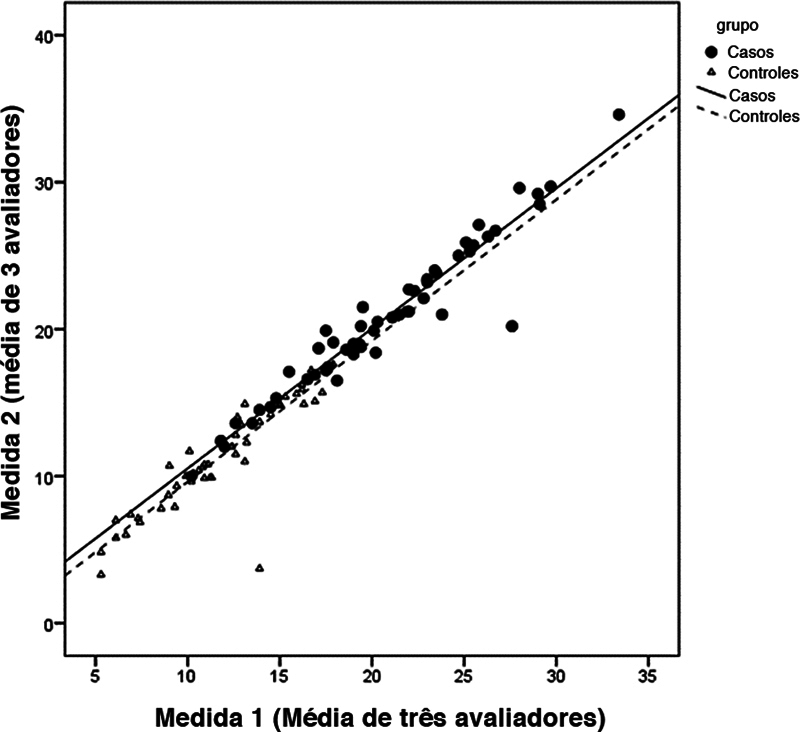
Avaliação da concordância intraexaminador.

A concordância intraexaminador apresentou boa relação entre as duas medidas feitas pelos três examinadores. O CCI foi de 0,99 no grupo instabilidade e 0,98 no grupo controle. A Fig. 2 mostra os dados analisados.

**Fig. 3:**
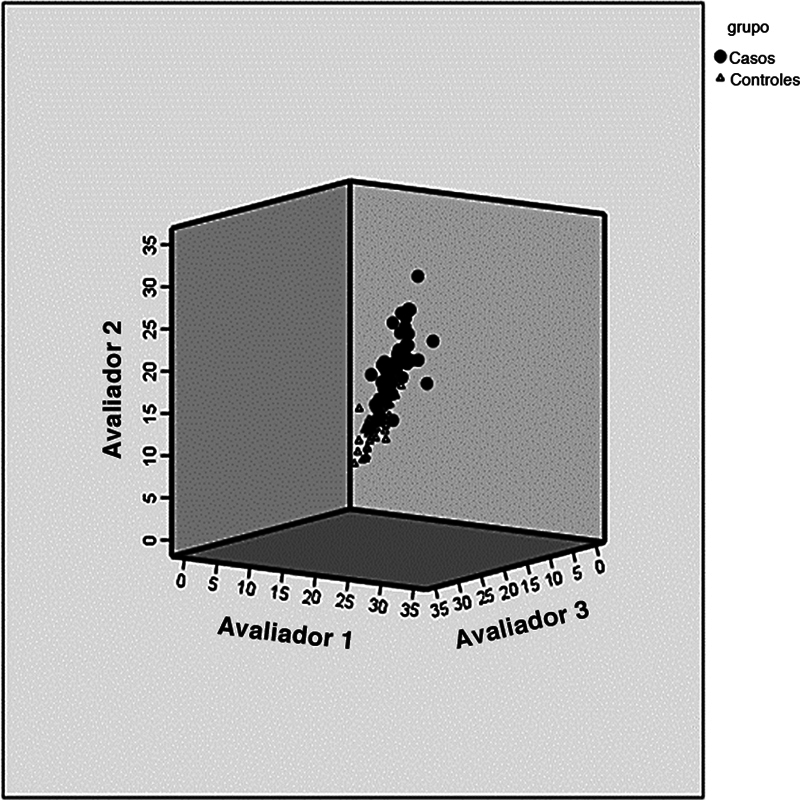
Avaliação da concordância interexaminadores.

Não houve diferença significativa nos valores encontrados pelos examinadores no grupo instabilidade. No grupo controle, o examinador 3 apresentou médias ligeiramente menores que o examinador 2. Apesar disso, o CCI foi de 0,98 em ambos os grupos, mostrando boa concordância interexaminadores ( Fig. 3 ).

**Fig. 4:**
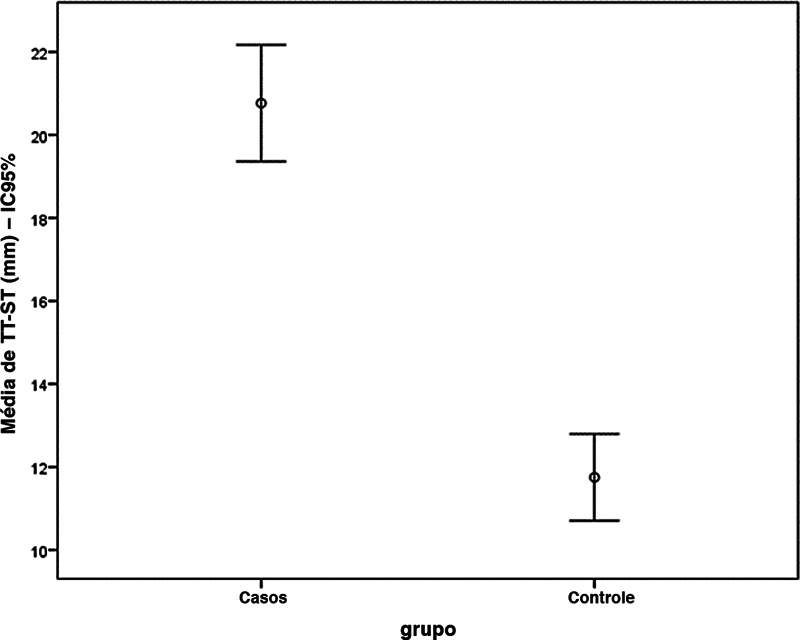
Comparação da medida tuberosidade tibial-sulco troclear (TT-ST) entre os grupos. **Abreviatura:** IC95%, intervalo de confiança de 95%.

Objetivamente, em termos do valor de TP-ST, observou-se uma média maior no grupo instabilidade em comparação ao grupo controle. No primeiro, a média foi de 20,6 ± 5,0 mm, enquanto a média no segundo grupo foi de 11,8 ± 3,4 mm ( *p* < 0,001). A comparação é ilustrada na Fig. 4 .

**Fig. 5:**
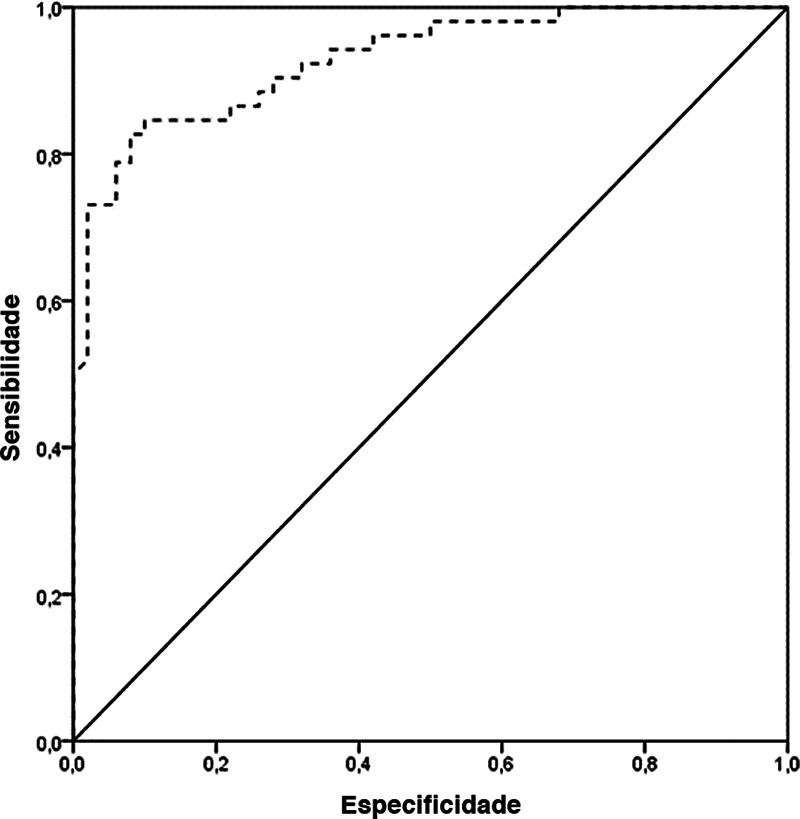
Curva característica de operação do receptor (ROC, do inglês *receiver operating characteristic* ) para determinação do melhor limiar da medida da distância tendão patelar-sulco troclear (TP-ST) na triagem de instabilidade patelar.

Por fim, o limiar patológico definido como um novo ponto de corte para medida da distância TP-ST à RM é 15,5 mm. Este limiar foi determinado pela curva ROC, com sensibilidade de 82,7%, especificidade de 80,0%, valor preditivo positivo de 81,1%, valor preditivo negativo de 81,6% e acurácia de 81,4%. Este ponto de corte é consistente como variável de rastreamento, como mostra a Fig. 5 .

## Discussão

Os principais achados deste estudo são que os pacientes com instabilidade patelar lateral apresentam uma medida maior de TP-ST em comparação a indivíduos sem instabilidade. Além disso, o valor de 15,5 mm de TP-ST em exames de RM representa um limiar patológico relacionado à instabilidade patelar lateral.

O vetor de força lateralizado do mecanismo extensor do joelho é um dos principais fatores de instabilidade patelar. A TC ainda pode ser considerada o padrão ouro para sua avaliação. No entanto, tornou-se discutível com a maior disponibilidade de exames mais específicos e sensíveis. No contexto atual, a RM tem enorme importância e deve ser usada para diagnósticos e planejamentos cirúrgicos. A RM permite a avaliação da cartilagem articular femoral e patelar, a identificação precisa da inserção do tendão patelar e a integridade dos ligamentos patelofemorais. Além disso, pode evitar a exposição à radiação e o custo adicional da TC.

A definição de um limiar patológico de 15,5 mm na medida do TP-ST na RM é um fator importante para se considerar a necessidade de osteotomia da TAT. Dessa forma, a RM deve ser considerada o único exame complementar necessário, além da radiografia do joelho, para diagnóstico da instabilidade patelofemoral e planejamento cirúrgico para seu tratamento.

Com a maior disponibilidade de exames de imagem detalhados, como na RM, agrega-se qualidade ao diagnóstico pré-operatório. Ao considerar partes moles e superfícies articulares, a RM tem maior definição que a TC. Embora a medida tomográfica seja o padrão-ouro para definição da distância TT-ST, estudos mostraram uma confiança interobservador menor que 60% nas medidas feitas com esse método. A maior dificuldade enfrentada foi determinar o ponto mais profundo do ST, principalmente na tróclea de joelhos com displasia excessiva. 14

Assim, corroborando estudos anteriores, 8 15 é possível encontrar excelentes taxas de confiabilidade inter e intraobservador para a medida da distância TP-ST em joelhos com luxação prévia ou sem instabilidade, com CCI elevado, de 0,98 e 0,99, respectivamente. Acredita-se que essa medida seja mais fisiológica em relação aos pontos de referência ósseos de uma TC, pois representa de fato o sítio de referência onde atuam as forças responsáveis pelo alinhamento do mecanismo extensor do joelho. 8 16

Camp et al. 7 analisaram as distâncias TT-ST e TP-ST em joelhos com instabilidade patelofemoral por TC e RM e relataram que ambos os métodos têm excelente confiabilidade interobservador. Os valores da distância TP-ST à RM foram constantemente menores, com uma diferença maior nos casos em que a medida de TT-ST por TC foi superior a 20 mm em joelhos displásicos. Portanto, esses resultados sugerem que a medida da distância TP-ST por RM é válida, mas os valores dos dois métodos de imagem não são intercambiáveis em pacientes com instabilidade

Pandit et al., 6 em um estudo com articulações de joelho clínica e artroscopicamente normais, encontraram valores médios de 10 ± 1 mm à RM, inferiores aos propostos por Dejour 4 para joelhos assintomáticos à TC. Da mesma forma, Thakkar et al. 17 observaram valores médios menores à RM do que à TC em joelhos sem instabilidade.

Em relação aos joelhos com instabilidade patelofemoral, Skelley et al. 15 encontraram valores de 18,2 mm à RM, novamente menores que os observados à TC em estudos anteriores. Esse padrão foi seguido em estudos posteriores, 18 19 20 ou seja, a medida da distância TP-ST de joelhos instáveis e não acometidos à RM apresentou valores menores em comparação à distância TP-ST medida por TC.

Poucos estudos compararam joelhos com e sem instabilidade quanto ao TP-ST medido à RM; assim, é necessário validar um novo limiar patológico para essa variável nesse tipo de exame. O presente estudo encontrou um ponto de corte de 15,5 mm e, com alta sensibilidade e especificidade, esse valor se mostrou uma ferramenta consistente como variável de rastreamento.

Neste estudo, os grupos diferiram em sua representação de gênero. No entanto, é importante ressaltar que os autores não consideraram o gênero no momento da seleção da amostra e da randomização já que não há diferença entre os gêneros nos valores médios de medidas de TT-ST e TP-ST descritos na literatura. 6 21

Vale ressaltar que este é um estudo de caso-controle que utilizou exames já realizados. O protocolo seguido para posicionamento do paciente para RM do joelho foi aquele utilizado na maioria das instituições: membro em rotação neutra e extensão completa sem contração do quadríceps. Além disso, mesmo havendo randomização dos controles, houve pacientes que haviam sido submetidos a exame de imagem devido a alguma queixa prévia não relacionada à articulação patelofemoral; portanto, não são imagens de articulações do joelho completamente assintomáticas.

Indivíduos com instabilidade patelar lateral apresentam uma medida estatisticamente maior de TP-ST do que pacientes sem tal instabilidade. Além disso, valores maiores de TP-ST superiores a 15,5 mm em exames de RM representam uma força de lateralização patológica do mecanismo extensor, que está relacionada à instabilidade patelar.
